# A Digital Intervention for Capturing the Real-Time Health Data Needed for Epilepsy Seizure Forecasting: Protocol for a Formative Co-Design and Usability Study (The ATMOSPHERE Study)

**DOI:** 10.2196/60129

**Published:** 2024-09-19

**Authors:** Emily E V Quilter, Samuel Downes, Mairi Therese Deighan, Liz Stuart, Rosie Charles, Phil Tittensor, Leandro Junges, Peter Kissack, Yasser Qureshi, Aravind Kumar Kamaraj, Amberly Brigden

**Affiliations:** 1 School of Engineering Mathematics and Technology University of Bristol Bristol United Kingdom; 2 School of Computing Ulster University Belfast Ireland; 3 Neuronostics Bristol United Kingdom; 4 The Royal Wolverhampton NHS Trust Wolverhampton United Kingdom; 5 Centre for Systems Modelling & Quantitative Biomedicine (SMQB) The University of Birmingham Birmingham United Kingdom; 6 School of Mathematics The University of Birmingham Birmingham United Kingdom; 7 School of Engineering The University of Warwick Coventry United Kingdom

**Keywords:** epilepsy, seizure forecasting, data science, artificial intelligence, machine learning, wearable technology, mobile phone

## Abstract

**Background:**

Epilepsy is a chronic neurological disorder affecting individuals globally, marked by recurrent and apparently unpredictable seizures that pose significant challenges, including increased mortality, injuries, and diminished quality of life. Despite advancements in treatments, a significant proportion of people with epilepsy continue to experience uncontrolled seizures. The apparent unpredictability of these events has been identified as a major concern for people with epilepsy, highlighting the need for innovative seizure forecasting technologies.

**Objective:**

The ATMOSPHERE study aimed to develop and evaluate a digital intervention, using wearable technology and data science, that provides real-time, individualized seizure forecasting for individuals living with epilepsy. This paper reports the protocol for one of the workstreams focusing on the design and testing of a prototype to capture real-time input data needed for predictive modeling. The first aim was to collaboratively design the prototype (work completed). The second aim is to conduct an “in-the-wild” study to assess usability and refine the prototype (planned research).

**Methods:**

This study uses a person-based approach to design and test the usability of a prototype for real-time seizure precipitant data capture. Phase 1 (work completed) involved co-design with individuals living with epilepsy and health care professionals. Sessions explored users’ requirements for the prototype, followed by iterative design of low-fidelity, static prototypes. Phase 2 (planned research) will be an “in-the-wild” usability study involving the deployment of a mid-fidelity, functional prototype for 4 weeks, with the collection of mixed methods usability data to assess the prototype’s real-world application, feasibility, acceptability, and engagement. This phase involves primary participants (adults diagnosed with epilepsy) and, optionally, their nominated significant other. The usability study will run in 3 rounds of deployment and data collection, aiming to recruit 5 participants per round, with prototype refinement between rounds.

**Results:**

The phase-1 co-design study engaged 22 individuals, resulting in the development of a mid-fidelity, functional prototype based on identified requirements, including the tracking of evidence-based and personalized seizure precipitants. The upcoming phase-2 usability study is expected to provide insights into the prototype’s real-world usability, identify areas for improvement, and refine the technology for future development. The estimated completion date of phase 2 is the last quarter of 2024.

**Conclusions:**

The ATMOSPHERE study aims to make a significant step forward in epilepsy management, focusing on the development of a user-centered, noninvasive wearable device for seizure forecasting. Through a collaborative design process and comprehensive usability testing, this research aims to address the critical need for predictive seizure forecasting technologies, offering a promising approach to improving the lives of individuals with epilepsy. By leveraging predictive analytics and personalized machine learning models, this technology seeks to offer a novel approach to managing epilepsy, potentially improving clinical outcomes, including quality of life, through increased predictability and seizure management.

**International Registered Report Identifier (IRRID):**

DERR1-10.2196/60129

## Introduction

### Background of the Study

Epilepsy, a chronic neurological disorder, is characterized by recurrent and unpredictable seizures, affecting individuals across all age demographics [1]. As a leading cause of chronic morbidity globally, epilepsy presents significant challenges, including a heightened risk of premature death, injuries, and diminished quality of life [2]. With a prevalence rate of 5-10 cases per 1000 people [1], the disorder imposes a considerable burden on individuals and health care systems alike. The financial impact is also notable, with the UK National Health Service spending approximately £1.5 billion (US $2 billion) annually on care that ranges from primary treatment and management to addressing seizure-related injuries and mental health issues [3,4]. The disability-adjusted life-years rates in the United Kingdom, estimated at 92,400 per 100,000 population [5], highlight the extensive health loss attributed to epilepsy. Sudden unexpected death in epilepsy remains a critical concern, with an incidence rate of approximately 1 in 1000 people with epilepsy annually, emphasizing the need for ongoing research and innovative management strategies [6,7]. Accurate forecasting of seizures could allow people with epilepsy to take additional safety precautions at times of increased risk.

### Rationale for the Study

Despite advancements in treatment—such as resective surgery, neurostimulation devices, new antiseizure medication, many with novel modes of action, not to mention the increasing availability of treatment across the world, with several antiseizure medications on the World Health Organization’s list of essential medicines—approximately one-third of the population (more than 50 million people) living with epilepsy globally still experience uncontrolled and apparently unpredictable seizures [8]. A large community survey (n=1056) [5] and the UK Epilepsy Priority Setting Partnership [9] ranked the unpredictability of seizures as one of the most impactful aspects of the condition and a research priority, underscoring an urgent need for solutions that can offer predictability and management of seizure risk. Seizure forecasting technologies emerge as a promising avenue in this regard, potentially enabling individuals to take pre-emptive measures during high-risk periods, therefore mitigating the risk of injury and premature death, and alleviating the psychological burden associated with the unpredictability of seizures [10].

One potential avenue for seizure forecasting is leveraging technologies that directly monitor brain activity, combining this with data analytics to explore seizure risk modeling. Video–electroencephalography (EEG) is the gold standard for monitoring brain biosignals to detect seizure activity. However, this is not well-suited to explore as an out-of-clinic seizure forecasting solution, as it is not feasible for long-term use in real-world scenarios [11]. Ultra–long-term electroencephalography monitoring (UNEEG) represents a significant advancement in the field [12]. UNEEG’s technology, involving surgical implantation, offers continuous EEG data capture in the user’s real-world context. People with epilepsy who have trialed the technology have found the continual monitoring valuable, both in terms of internalizing their locus of control and providing information to inform treatment changes. However, UNEEG is a minimally invasive method with intrinsic risks and costs of surgery, making it inaccessible to a wider range of users [12,13]. Noninvasive scalp EEG represents a more accessible solution that is designed for use outside of clinical settings. Yet, the adoption of this technology is hampered by limitations such as the stigma of visible devices, practicality, acceptance among users [13], and the technical and practical challenges of long-term outpatient monitoring [14].

An alternative seizure forecasting approach is to explore predictive analytics based on seizure precipitants that can be captured through noninvasive, nonmedical grade wearable devices (WDs), such as smartwatches. This presents a high-potential solution, as ubiquitous wearable technologies can be easily integrated into the end user’s everyday life, to continuously capture real-time data over the long term. Noninvasive WDs that can be used outside of clinical settings have been successfully used for epilepsy management in the form of the Empatica E4 wristband [11]. This device has shown promise in detecting epileptic seizures by monitoring physiological parameters such as heart rate and acceleration. However, the Empatica E4 focuses primarily on seizure detection rather than forecasting [11].

Exploration of seizure forecasting based on seizure precipitant data remains in its infancy. Emerging research has explored seizure cyclicity for forecasting models. However, these approaches on their own have limited accuracy for seizure forecasting [15-17]. There is potential to build upon and improve these models by including a greater range of seizure precipitants. This is a promising approach, given the existing evidence on the range of external and physiological variables that play a precipitating role in seizure propagation. Measurable biomarkers, such as emotional stress, infections, menstrual cycles, sleep deprivation, and alcohol use, have all been identified as seizure precipitants. The relationship between precipitants and seizure propensity could be quantified using mathematical models [13,17], with these models informing algorithms to analyze person-specific data and facilitating personalized seizure forecasts.

Ubiquitous technologies with multimodal sensors, combined with ecological momentary assessment (EMA; repeated sampling of an individual’s cognitions, emotions, symptoms, and behaviors) could enable the real-time capture of the broad spectrum of known seizure precipitants. These real-time data, combined with predictive analytics, offer a feasible and accessible solution for seizure forecasting. To our knowledge, there is a pressing need to address the research gap in developing and evaluating technology for real-time seizure precipitant data collection, essential for supporting seizure forecasting interventions.

### Person-Based Approach

A technology to collect real-time seizure precipitant data relies on people with epilepsy actively engaging with the technology. As such, it is essential that a user-centered design approach is taken. The person-based approach [18] integrates methods from user-centered design and qualitative research to design and evaluate complex digital health technologies. This approach can lead to solutions that are more acceptable, feasible, meaningful, and optimally engaging to the people who will use them. The person-based approach advocates undertaking iterative design cycles with users through both formal qualitative research and patient and public involvement. Previous research [16,19] underscores the necessity of using person-based approach for the future design of epilepsy-related technologies, emphasizing iterative design cycles and user involvement to create solutions that are not only technologically effective but also deeply aligned with the needs and experiences of those living with epilepsy. Therefore, this study used the person-based approach, recognizing its critical role in developing effective, user-centered epilepsy management technologies.

The ATMOSPHERE study, funded by the Engineering and Physical Sciences Research Council (EPSRC) and in collaboration with the N-CODE network [20], represents an effort to leverage technology for the benefit of individuals with epilepsy. The ATMOSPHERE project is an early-stage, proof-of-concept research initiative seeking to develop and evaluate a noninvasive, wearable, epileptic seizure forecasting technological solution. This innovative approach aims to tackle the apparently unpredictable nature of seizures by collecting real-time data on various seizure precipitants, such as sleep quality, heart rate, menstrual cycle, medication adherence, and stress levels, from adults living with epilepsy.

### The ATMOSPHERE Project

The entire project is organized into several work packages, each targeting a specific aspect of seizure forecasting technology development, such as (1) work package 1—synthesizing the evidence based on seizure precipitants or triggers, (2) work package 2—designing and testing the usability of a mid-fidelity functional prototype to capture real-time precipitant data from individuals living with epilepsy, (3) work package 3—developing predictive models for seizure risk forecasting, and (4) work package 4—identifying user requirements for the end user app interface including presenting seizure forecasting output. Developing a high-fidelity functional prototype is based on the outcome of the usability study in work package 2.

This protocol paper specifically addresses workstream 2 of the ATMOSPHERE study, reporting the results from the completed co-design study and outlining the protocol for the upcoming usability study.

### Aims of Work Package 2

This multiphase research workstream aims to design, develop, and test prototypes for collecting real-time data on seizure and seizure precipitants.

### Objectives

The first objective is to collaboratively design a mid-fidelity functional prototype capable of collecting real-time seizure and seizure precipitant data from people with epilepsy. The second objective is to conduct “in-the-wild” usability testing of this prototype to evaluate its feasibility and user acceptance and engagement. This step is critical to assessing the data capture prototype’s real-world application and identifying areas for improvement based on user feedback. The third objective is to use insights gained from usability testing to refine and enhance the prototype, and focus on improving its functionality, ease of use, and overall effectiveness in collecting high-quality data for seizure forecasting.

## Methods

### Study Design

**Figure 1:**
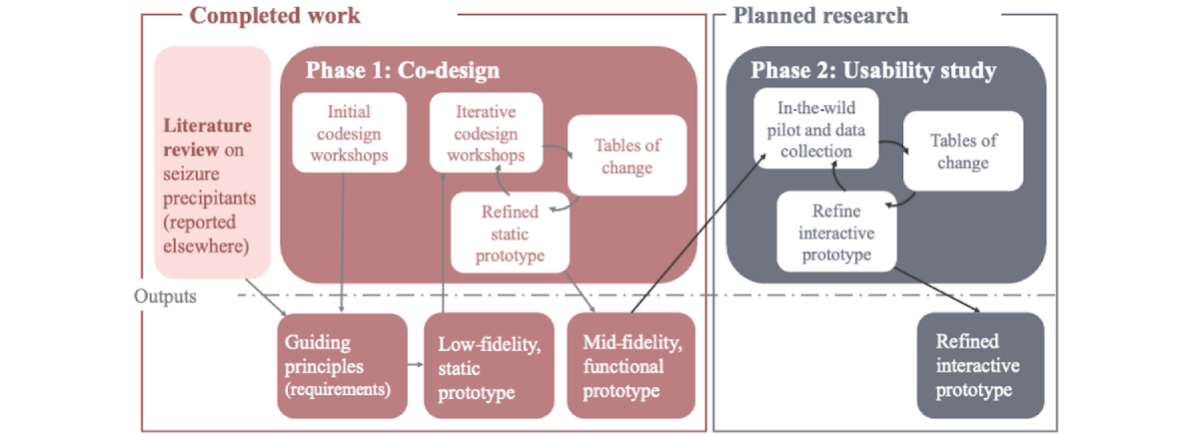
Overview of study phases, with the study design based on the person-based approach.

Drawing on the person-based approach [21], we undertook iterative patient and public involvement to co-design the data collection prototype (phase 1). We are now preparing to conduct a mixed methods “in-the-wild” usability study in phase 2 to further investigate the prototype’s usability and implement refinements (Figure 1).

### Phase 1: Co-Design—Completed Work

#### Co-Design Contributors

##### Lived Experience Contributors

We disseminated an advertisement for the co-design sessions through Epilepsy Action (a UK epilepsy charity) and the SHAPE Network (the Epilepsy Research Institute; United Kingdom’s patient and public involvement and engagement service group). The advertisement invited individuals with lived experience of epilepsy to take part in the co-design workshops and provided a link to an expression of interest form capturing consent for the research team to contact the individual. We invited individuals completing the expression of interest form to complete a form capturing clinical and demographic characteristics; these data were pseudonymized and used to understand the diversity of the group.

##### Health Care Professional Contributors

We used our existing professional networks to invite health care professionals with experience in providing care for epilepsy, both in primary and secondary care services.

#### Co-Design Sessions

##### Lived Experience Contributor Sessions

The co-design involved iterative workshops, based on workshop plans and lasted 60-90 minutes. Early sessions explored the acceptability of collecting evidence-based precipitant data, views on the content of EMA items, the acceptability of different wearables for data collection, and the wider sociotechnical space within which the technology would be used. From these early sessions, a guiding principles document [21] was developed to identify the users’ needs, context, and required components for the data-collection technology (presented in *Phase 1: Co-Design—Completed Work* in the *Results* section). Based on the guiding principles, we developed a low-fidelity prototype in Microsoft PowerPoint. In subsequent user consultation sessions, we iteratively refined the prototype by gathering feedback, completing a table of changes document (a summary of required changes) [21], and implementing required changes.

##### Health Professional Contributor Sessions

We conducted one-to-one discussions through video calls using a semistructured topic guide lasting 30-60 minutes. Discussions explored the health professionals’ views on design considerations from the perspectives of people with epilepsy, design considerations from the clinician’s perspective (eg, how data might fit into the clinical workflow), and an investigation into the potential harms or unintended consequences. From these discussions, a guiding principles document was developed that informed prototype design.

#### Prototype

**Figure 2:**
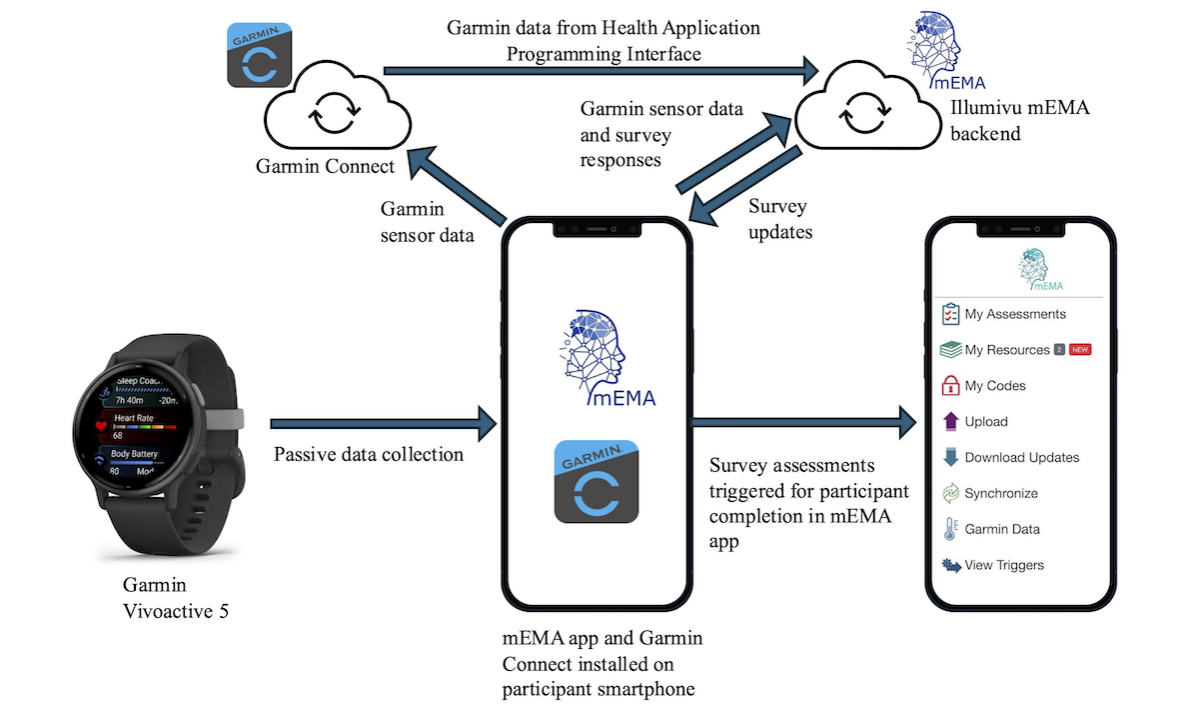
An overview of the mid-fidelity, interactive prototype for the primary participant. API: application programming interface.

At the end of the co-design process and in preparation for the usability study, a functional mid-fidelity prototype was developed (discussed in detail in the *Results* section; Figure 2).

### Phase 2: Usability Testing—Protocol for Planned Research

#### Participants

##### Primary Participants

The primary participants in this study will be adults diagnosed with epilepsy. We will disseminate an advertisement through Epilepsy Action, the SHAPE Network, and Caafi Health (an organization with expertise in engaging underserved communities, for example, those from ethnic minority groups) [22]. The inclusion and exclusion criteria for participation in the study are detailed in Textbox 1. These criteria were established to ensure that participants will be representative of the broader population of individuals with epilepsy, while also considering safety and ethical considerations pertinent to conducting research within this group.

**Inclusion and exclusion criteria.**

**Inclusion criteria**

- Primary participant (participant with epilepsy diagnosis)
  Adult (older than 18 years)
  Have a diagnosis of epilepsy
  Likely to have a seizure within the 4-week testing period (based on self-report)
  Are fluent in English (to be able to engage with the technology and qualitative interview)
  Can provide consent
  Can wear a wrist wearable device (smartwatch)
  Able to input data into a smartphone app
  Own a smartphone that can download apps from either the Apple Store (iPhone) or the Google Play Store (Android phones; individuals were not required to own a smartwatch as this was supplied as part of the study)
- Nominated participants (a significant other identified by the primary participant)
  Nominated by a person meeting the criteria above
  Adult (older than 18 years)
  Fluent in English (to be able to engage with the technology and qualitative interview).
  Able to consent to the study
  Own a smartphone which can download apps from the Google Play Store or the Apple Store
  Be able to input data into a smartphone app

**Exclusion criteria**

- Primary participant (participant with epilepsy diagnosis)
  Those who have a major cardiac condition
  Those with a diagnosis of a major psychiatric condition (defined as needing secondary care mental health services support)

##### Nominated Participants

The primary participants (adults living with epilepsy) will be invited to nominate a significant other, such as a carer, friend, or family member, to also participate in the study. This will be optional. The inclusion and exclusion criteria for nominated participants are detailed in Textbox 1.

#### Recruitment and Consent Process

##### Primary Participants (Individuals Living With Epilepsy)

Potential participants will be given a participant information sheet and invited to contact the research team to express interest in the study. Those expressing interest will be asked to complete a sampling form, providing demographic and clinical information; this will be used to undertake purposeful sampling, aiming to recruit a diverse sample in terms of age, gender, ethnicity, and the type of epilepsy. Individuals sampled for the study will initially be contacted by an epilepsy clinician who will screen for eligibility. Eligible participants will then be contacted by a member of our research team for a recruitment consultation. Following the recruitment and screening consultation, informed consent will be obtained from individuals wishing to participate. This consent process will be conducted in accordance with ethical guidelines to ensure that participants are fully informed and willing to participate in the study voluntarily.

##### Nominated Participants

On entering the study, the primary participants will be invited to nominate a significant other (eg, family member, friend, and carer) and provide consent for us to contact this individual. We will contact these nominated individuals, provide them with a participant information sheet, undertake a recruitment consultation, and obtain fully informed consent from those wishing to take part.

#### Sampling and Sample Size

To achieve a comprehensive understanding of the usability and effectiveness of the intervention, we aim to recruit approximately 15 primary participants (with additional nominated participants). Recruitment will be organized into 3 rounds, with each round consisting of 5 participants. The sample size of 5 primary participants per round was based on the usability testing literature, where a sample size of 3-5 participants has been found to detect the majority of usability problems [23]. This phased approach will allow for iterative refinements to the intervention and study procedures based on feedback from each round of participants, enhancing the robustness and relevance of our findings.

#### Study Procedures

##### Participant Procedure 1: Customization Survey and Onboarding

The prototype is a smartwatch and smartphone app for the primary participant and a smartphone app only for the nominated participant (full description of the prototype is given in the *Methods* section). Participants will be sent a questionnaire (using Microsoft Forms) to gather their requirements for customization (description of customization options is given in the *Methods* section), and the prototype will then be adapted to their requirements. Participants will be sent the prototype, along with details for downloading the smartphone app, and we will undertake an onboarding meeting to orientate participants to the technology.

##### Participant Procedure 2: Deployment of Data Collection Prototype

Participants will be invited to use the technology for 4 weeks. The 4-week study period was intentionally chosen to encompass a complete menstrual cycle, enabling comprehensive capture of physiological data parameters across the full cycle. If the research team notices that the user has not worn the device or manually entered the data, the participant will be sent an email offering a technical support meeting.

##### Participant Procedure 3: Qualitative Interview

At the end of the 4-week usability period, the participants will be invited to a qualitative interview with a member of the research team (video call or face-to-face, depending on participant preference and feasibility of travel for the research team). This qualitative interview will use a semistructured topic guide, will be designed to last up to 60 minutes, and will be audio recorded by Microsoft Teams. We will explore various fields, such as section 1, an open-ended exploration of the participant’s views and experiences of the technology, including an exploration of the technology within the sociotechnical space; section 2, exploration of the technology features, for example, cotracking and passive data collection; section 3, exploration of the specific items and wording of the EMA; and section 4, exploration of views to improve the technology (also gathered throughout sections 1-3)

Upon completion of the study, participants will receive financial compensation and can opt to receive a visual summary of the data collected.

#### Outcome Measures to Investigate Feasibility, Acceptability, and Engagement

The outcome measures being used to determine feasibility, acceptability, and engagement are reported in Table 1.

**Table 1:** Outcome measures for feasibility, acceptability, and engagement.

Outcome measure			Data points		Analysis
**Quantitative outcome measures**					
	Smartwatch wear time	Assessed using the presence of the heart rate data		Descriptive statistics^a^ on Proportion of days the device was worn for Number of hours during the day the device was worn for	
	EMA^b^ response rate	Assessed for each EMA item		Descriptive statistics on response rate for each EMA item	
**Qualitative outcome measure**					
	Users’ perspectives	Qualitative interview data		Thematic analysis [24] on pseudonymized transcripts to identify issues related to acceptability, feasibility, and engagement Tables of change to identify requirements to optimize the prototype	

^a^Descriptive statistics: measures of central tendency (mean and median) and dispersion (SD and IQR) to provide a comprehensive overview of the wear time, response rate, and missing data.

^b^EMA: ecological momentary assessment.

#### Prototype Refinement

After each usability round, we will analyze qualitative interview data to produce a table of changes document, identifying requirements to optimize the prototype [21]. If feasible, these changes will be implemented before the next round of deployment (Figure 1).

### Ethical Considerations

Phase 1 involved a co-design with patient and public involvement contributors, which does not necessitate formal ethical approval [25]. However, we adhered to the General Data Protection Regulation (GDPR) guidelines [26]. To protect patient and public involvement contributor privacy, we used pseudonymization for sampling forms, and we secured consent to report aggregated data. During co-design sessions, no direct quotes were captured; instead, we created summaries in the form of guiding principles and tables of changes. We offered patient and public involvement contributors the equivalent of £20 per hour (US $26) in shopping vouchers.

For phase 2, ethical approval was obtained from the University of Bristol, Faculty of Engineering Research Ethics Committee on March 10, 2022 (reference 10152). In alignment with our commitment to data privacy and protection, we carried out a data protection impact assessment [27]. This assessment (ensures compliance with the GDPR within the European Union [26]), aims to evaluate and minimize potential risks to the rights and freedoms associated with personal data. The information compliance manager and data protection officer from the University of Bristol approved the data protection impact assessment on January 29, 2024.

## Results

### Phase 1: Co-Design—Completed Work

#### Overview

We involved 12 individuals with lived experience of epilepsy, undertaking 21 consultations across the iterative design process. We involved 10 clinicians, with each individual contributing once. The demographic and clinical demographics are presented in Table 2. Table 3 presents the guiding principles derived from the co-design sessions. The first row is based on existing evidence, the subsequent rows are based on the co-design.

**Table 2:** Summary of numbers, demographic, and clinical characteristics of patient and public involvement contributors.

Characteristics				n (%)
**Individuals with lived experience (n=12)**				
	**Gender**			
		Women	8 (67)	
		Men	4 (33)	
	**Geographical region**			
		South East England	7 (58)	
		South West England	3 (25)	
		West Midlands	1 (8)	
		East Midlands	1 (8)	
	**Ethnicity**			
		White British	10 (83)	
		Not reported	2 (17)	
	**Age (years)**			
		More than 65	1 (8)	
		55-64	2 (17)	
		45-54	2 (17)	
		35-44	4 (33)	
		25-34	1 (8)	
		Not reported	2 (17)	
	**Levels of deprivation^a^**			
		10% and 20% most deprived	0 (0)	
		30% and 40% most deprived	4 (33)	
		50% most deprived	1 (8)	
		50% least deprived	3 (25)	
		30% and 40% least deprived	4 (33)	
		10% and 20% least deprived	0 (0)	
	**Seizure types^b^, n**			
		Tonic clonic	5	
		Focal impaired awareness	4	
		Absence	3	
		Complex partial	2	
		Focal aware	2	
		Tonic	1	
		Atonic	1	
		Myoclonic	1	
		Drop seizures	1	
		Not reported	3	
**Health professionals (n=10)**				
	**Professional background**			
		General practitioner	3 (30)	
		Epilepsy nurse specialist	3 (30)	
		Learning disability nurse or psychiatrist	2 (20)	
		Junior doctor	2 (20)	

^a^Estimated using contributors’ postcodes against the 2019 English Indices of Deprivation data.

^b^Some contributors identified as having multiple characteristics within the domain.

**Table 3:** Guiding principles for data collection technology.

User characteristics			Key feature(s)^a^
**Individuals with epilepsy**			
	Individuals with epilepsy are likely to have precipitants to seizures, as documented in the evidence.	Features to enable tracking of a range of evidence-based precipitants: emotional states, stress, fatigue, sleep disturbance, phase of menstrual cycle, seizure cycles, medication usage, and fever or illness.	
	In addition to evidence-based precipitants, individuals experience: A wide range of other seizure precipitants (eg, cognitive overwhelm and certain foods). Auras and prodromal symptoms (eg, speech, emotional, and behavioral changes). Precipitants and prodromal symptoms can be personal (ie, specific to that individual), subjective (ie, hard to capture with objective, sensing data), and sometimes the individual can lack insight in these variables (see cotracking below).	Features to offer customized tracking of personal seizure precipitants and prodromal symptoms, including subjective experiences.	
	SUDEP^b^ is a major concern for users, and users would like SUDEP risk assessments. Some users already complete regular SUDEP risk assessments (including on mobile apps).	There are existing apps for SUDEP risk assessment [28]. Consideration should be given to how the apps could work together.	
	With assurances around privacy and security, individuals are positive about providing data that may improve forecasting performance. Most users are positive about active, manual tracking, viewing this as necessary for subjective data points (eg, mood and fatigue). Many users already actively track seizures and precipitants (eg, through paper diaries and spreadsheets). Some users described tracking as giving a sense of control and empowerment. However, active tracking can be a burden and challenging due to memory impairment and reduced insight. There may be strong individual differences (see “individual differences” below) in the desire or ability to actively track.	Features to maximize trust, identity, privacy, and security in the collection, processing, and storage of data. Features to passively track precipitants, prioritizing passive where possible. Features to enable manual tracking of trigger data, as an option for those willing and able.	
	Epilepsy management often involves carers, families or friends, and individuals with epilepsy would like the technology to reflect this. Some individuals with epilepsy are fully reliant on carers to track precipitants. Some manage independently but still have periods when they need support from their significant other(s) to track precipitants (eg, during periods of epilepsy-related amnesia or reduced consciousness, due to cognitive impairment and due to deceased personal insight). Individuals still want a sense of independence and control over the tracking process.	Features to enable optional cotracking; whereby significant others (family, friends, or carers) can also track data on the primary user’s health states. This feature must preserve independence, giving the user control over who tracks, what they track, and control over stopping cotracking.	
	Different seizure types present different types of risks to individuals and require different self-management approaches.	Feature for individuals to track different seizure types, to enable forecasting to differentiate between seizure types.	
	There are individual differences in the user group. Some that need particular attention are: The variability in the motivation for actively tracking data. Vulnerable and digitally excluded groups need careful consideration. This may include older adults (with a higher prevalence of certain types of epilepsy, for example, poststroke seizure), those with an intellectual disability (who have higher prevalence of epilepsy and treatment-resistant seizures), and those who are socially disadvantaged (eg, lower level of education, drug users, prisoner population, and homeless populations).	Simplicity and accessibility are essential. The technology should draw upon HCI^c^ principles to achieve inclusive design [29].	
**Clinicians (secondary care)**			
	Out-of-clinic data are critical for diagnosis and treatment planning. However, out-of-clinic data are often limited or can be inaccurate. Fuller and accurate data that would be useful for diagnosis and management include: seizure data: type, frequency, and duration postictal symptoms medication adherence medication side effects the individual precipitants	Features to enable tracking of data useful for clinical management.	

^a^Key feature(s): a feature is a component of an application from the end user’s perspective.

^b^SUDEP: sudden unexpected death in epilepsy.

^c^HCI: human-computer interaction.

#### Prototypes and Tables of Change

Based on the guiding principles, a low-fidelity prototype was initially developed, and a table of changes was used to capture user requirements for further refinement. Based on the final tables of change, a mid-fidelity and functional prototype was developed (presented in Figure 2 and Table 4). The mid-fidelity functional prototype and study materials (customization questionnaire and onboarding process) were tested with 3 patient and public involvement contributors (all people with epilepsy) before the implementation of the main usability study.

**Table 4:** Data collection protocol from primary participants.

Data point			Data collection schedule
**Garmin (passive data)**			
	Sleep score	Daily summary	
	Heart rate	Continuous	
	Respiration rate	Continuous	
	Pulse oximetry blood oxygen saturation	Continuous	
	Stress score	Continuous	
	Accelerometer	Continuous	
	Ambient light	Continuous	
	Step count	Continuous	
**Smartphone App (EMA^a^)**			
	Seizure event (type, timing, duration, and severity)	Notification^b^: twice daily User-initiated^c^	
	Prodromal symptoms	Notification: once daily	
	Postictal symptoms	Notification: once, if seizure event reported	
	Epilepsy medication usage (routine and emergency)	Notification: once daily notification User-initiated	
	Emotional states and emotional intensity	Notification: twice daily User-initiated Contingent^d^: if elevated stress detected on Garmin (capped at 3 notifications daily)	
	Sleep duration and quality	Notification: once daily	
	Acute illness or infection	Notification: once daily	
	Customizable precipitant question	Notification: once daily	
	Alcohol intake	Notification: once daily	
	Menstrual cycle	One-off series of questions	

^a^EMA: ecological momentary assessment.

^b^Notification: smartphone notification pushed to user as a prompt for them to complete the EMA item.

^c^User-initiated: users able to open the app and complete the EMA item any time.

^d^Contingent: a push notification prompting EMA completing sent, based on data from the Garmin device.

#### Primary Participant

Data collection will take place through the mEMA (Illumivu) app installed on participants’ smartphones, connected to a Garmin VivoActive 5 smartwatch worn by participants as outlined in Figure 2. Quantitative data captured passively through the Garmin wearable (Table 4) will be streamed to the mEMA app and relayed to an encrypted database hosted by Illumivu. Simultaneously, Garmin sensor data will be relayed to Garmin Connect and processed through Garmin’s algorithms to generate compound measures (based on a combination of variables), such as stress level and sleep score, which will be streamed to Illumivu’s backend by Garmin’s Health application programming interface (Figure 2). The use of Garmin’s API enables access to the raw data from the smartwatch. These data are transmitted by Bluetooth directly from the WD to the mEMA app on the phone. From there, raw data are sent up to the Illumivu servers to be integrated with incoming EMA data.

The EMA surveys that participants will receive in the mEMA app have been developed for participants to record data on seizures (type, timing, duration, and severity), prodromal symptoms, postictal symptoms, and seizure precipitants (triggers). There will be three modes for EMA completion: (1) daily notifications to the participant at a set time to prompt EMA survey completion; (2) contingent notifications, based on Garmin data (eg, elevated stress levels) to prompt survey completion; and (3) user-initiated data entry, where the users can access the app and enter the data on an ad hoc basis. Completed surveys will be uploaded to the secure mEMA backend where they can be accessed by permissioned members of the core research team.

#### Customizable Components

There are some elements that can be customized by the participants. First, EMA notification timings—EMA notifications will be sent between 8 AM and 8 PM. Users can indicate times within this window during which they do not wish to receive notifications. Second, optional survey items—users can opt-in to receive questions on the menstrual cycle and EMA items on alcohol usage. Third, personalized EMA item—users will be asked if they would like to track an individual, personal seizure precipitant, and if feasible, we will create a personalized EMA item to capture these data.

#### Nominated Participant for Cotracking (Optional)

During the customization process, the primary participant will select which EMA items they consent for the nominated participant to receive (options being medication usage, emotional states and intensity, sleep duration and quality, alcohol intake, seizure events, prodromal symptoms, and postictal symptoms). The nominated participant will use the mEMA app installed on their smartphone and will receive notifications as prompts to complete the EMA survey.

### Phase 2: Usability Testing—Planned Research

This phase focuses on usability and the refinement of the prototype. Data collection for phase 2 started in May 2024, and the findings are expected to be available in August 2024.

## Discussion

### Principal Findings

This paper reports (1) the co-design process of a novel technology designed to collect real-time data needed for seizure forecasting and (2) the protocol for the usability testing of the prototype. Our co-design identified guiding principles for the prototype, including:

1. The implementation of tracking features for both evidence-based and custom seizure triggers, sudden unexpected death in epilepsy risk data, and data to support clinical management. These features are designed to maximize user trust, identity protection, privacy, and security in the handling of data.
2. Where feasible and appropriate, passive tracking methods should be used, with the option for users to log data actively or manually on potential seizure precipitants.
3. The introduction of cotracking features, giving users the freedom and capability to invite significant others (such as caregivers, friends, or family members) to participate in tracking precipitants of seizure onset.
4. The accommodation of diverse user needs and preferences, ensuring the design is inclusive and considers individual differences.

### Strengths and Limitations

This project emphasized the co-design of the prototype, ensuring that the technology aligns with the preferences and requirements of its target community. We engaged with clinicians from a range of relevant clinical specialties. We also engaged with a diverse group of individuals living with epilepsy, varying in aspects such as gender, age, seizure type, and socioeconomic background (determined by indices of deprivation). Individuals living with epilepsy are a diverse group, and we acknowledge that it will be important to continue to gather more views on the technology beyond the 15 individuals involved in the phase-1 co-design. A notable limitation of phase 1 is the lack of ethnic diversity among contributors and absence of individuals from the very lowest indices of deprivation. To find a remedy, in phase 2, we are working with a specialized organization to actively engage and involve ethnic minority groups and those from more deprived areas. This approach aims to make the development more inclusive and the technology more representative of the community it serves.

The second phase of our study is designed to evaluate the usability of our technology in a naturalistic setting (in the wild). The prototype undergoing testing is currently focused exclusively on the capabilities related to data collection. Consequently, it does not yet incorporate the advanced features, such as predictive analytics, for determining seizure risk scores and the provision of seizure forecasting outputs to end users. These more sophisticated functionalities are currently under development within separate workstreams. It is hypothesized that the availability of seizure forecasting output would serve as the primary motivation for individuals to continuously use the technology over an extended period. Therefore, the current limitation of the prototype, in its inability to replicate this aspect of real-world application and motivation, is acknowledged. To offset this limitation, we have endeavored to introduce an alternative form of motivation for participants by offering summaries of their data (data visualizations of seizure and precipitant data) at the conclusion of the study period.

The data collection protocol, including the type and frequency of prompts, was co-designed with individuals with lived experience. Subsequently, the prototype was tested with 3 patient and public involvement contributors who did not raise concerns about the frequency of prompts. Instead, users welcomed the reminders, especially those experiencing the well-documented memory impairments associated with epilepsy [30]. However, there is a possibility that the frequency of prompts could be burdensome when the technology is used in the wild over an extended period. During qualitative interviews, we will explicitly explore participants’ views on the data collection protocol, and we will refine the protocol, where necessary, based on this feedback.

The prototype developed for this phase is characterized as a mid-fidelity and functional prototype, which permits the testing of its core functionalities, such as passive data collection and EMA. Despite its capabilities, this prototype is not without its limitations, including a user experience interface that may not meet high standards and the necessity for some manual operations. However, the adoption of an agile methodology for this phase offers significant advantages. This approach allows for rapid learning and adjustments based on real-world, in-the-wild use, aligning with the principles of technological probing. While acknowledging its current constraints, this phase is pivotal in facilitating immediate, invaluable insights into the usability and potential impact of the technology in everyday contexts, guiding subsequent developments and enhancements.

This phase of the study did not include individuals with intellectual disabilities, an important population given their increased risk of epilepsy [31]. Recognizing the necessity of designing interventions that cater specifically to their needs, it is evident that this group requires a thorough investigation into their specialized design requirements (beyond the scope of this study). Such an exploration should be a priority for future research to ensure the development of more inclusive and effective solutions. Furthermore, the study focuses on adults, and a separate investigation for pediatric epilepsies is needed.

Overall, this work will inform the development of a high-fidelity functional prototype. The assessments of that prototype will be conducted through user experience walkthroughs and qualitative interviews to identify usability and accessibility challenges. The results from these upcoming assessments will be reported in a separate paper for work package 4.

### Conclusions

The co-design presented here identified user requirements for technology to capture the real-time data needed for seizure forecasting. Requirements include the ability to collect data passively where possible (with options for manual data input); collect custom, personal seizure precipitant data (in addition to evidence-based precipitant data); enable cotracking, where family or carers can input data; and consider inclusive design (due to variation in user characteristics and needs). The planned research in phase 2 will test the usability test of a mid-fidelity and functional prototype developed to meet these requirements. This co-design and usability testing contributes to a broader research aim; to develop seizure forecasting technology, leveraging real-time data on seizure precipitants, and using predictive analytics.

This technology could significantly improve the quality of life for individuals with epilepsy. By providing insights into the likelihood of seizure occurrence, such technology could offer a new level of independence and safety, reduce the economic and health care burdens associated with epilepsy, and facilitate a more personalized and efficient use of health care resources for epilepsy management. This technology development will be explored through proof-of-concept research of developing a noninvasive, WD specifically designed for seizure forecasting, integrating machine learning models for personalized predictive analytics, and using smartphone apps for real-time feedback to end users.

## Abbreviations

EEG: electroencephalography
EMA: ecological momentary assessment
EPSRC: Engineering and Physical Sciences Research Council
GDPR: General Data Protection Regulation
UNEEG: ultra–long-term electroencephalography monitoring
WD: wearable device

## Data Availability

Data sharing is not applicable to this article as no data sets were generated or analyzed during this study. Phase 1 was co-designed through patient and public involvement; therefore, no research data are available. Phase 2 is planned research; therefore, no research data are available.
